# Diagnostic properties of metabolic perturbations in rheumatoid arthritis

**DOI:** 10.1186/ar3243

**Published:** 2011-02-08

**Authors:** Rasmus K Madsen, Torbjörn Lundstedt, Jon Gabrielsson, Carl-Johan Sennbro, Gerd-Marie Alenius, Thomas Moritz, Solbritt Rantapää-Dahlqvist, Johan Trygg

**Affiliations:** 1 Computational Life Science Cluster (CLiC), Department of Chemistry, Umeå University, SE-90187 Umeå, Sweden; 2 Department of Medicinal Chemistry, BMC, Uppsala University, SE-75123 Uppsala, Sweden; 3 AcureOmics, Tvistevägen 48, SE-90736 Umeå, Sweden; 4 Active Biotech Research, Scheelevägen 22, SE-22007 Lund, Sweden; 5 Department of Public Health and Clinical Medicine, Rheumatology, Umeå University Hospital, Umeå, Sweden; 6 Umeå Plant Science Center, Swedish University of Agricultural Sciences, SE-90183 Umeå, Sweden

## Abstract

**Introduction:**

The aim of this study was to assess the feasibility of diagnosing early rheumatoid arthritis (RA) by measuring selected metabolic biomarkers.

**Methods:**

We compared the metabolic profile of patients with RA with that of healthy controls and patients with psoriatic arthritis (PsoA). The metabolites were measured using two different chromatography-mass spectrometry platforms, thereby giving a broad overview of serum metabolites. The metabolic profiles of patient and control groups were compared using multivariate statistical analysis. The findings were validated in a follow-up study of RA patients and healthy volunteers.

**Results:**

RA patients were diagnosed with a sensitivity of 93% and a specificity of 70% in a validation study using detection of 52 metabolites. Patients with RA or PsoA could be distinguished with a sensitivity of 90% and a specificity of 94%. Glyceric acid, D-ribofuranose and hypoxanthine were increased in RA patients, whereas histidine, threonic acid, methionine, cholesterol, asparagine and threonine were all decreased compared with healthy controls.

**Conclusions:**

Metabolite profiling (metabolomics) is a potentially useful technique for diagnosing RA. The predictive value was without regard to the presence of antibodies against cyclic citrullinated peptides.

## Introduction

Rheumatoid arthritis (RA) is an autoimmune disease in which the synovial tissues of the affected joints are invaded by cells of the immune system [1,2]. Primarily the hands and feet are affected, but other joints may be progressively involved. Patients with RA experience a progressive destruction of the affected joints, leading to disability. There have been substantial advances in the understanding of the disease pathogenesis, which has led to important improvements in the treatment of RA, most notably the introduction of biological disease modifiers such as tumor necrosis factor-α (TNF-α) inhibitors [3]. However, since the aetiology, apart from the identification of some genes associated with the disease, remains largely unknown, causal treatment is basically unavailable.

Even though the value of early intervention in RA has been appreciated for a long time, the diagnostic criteria applied for a diagnosis of RA are less appropriate in early disease [4,5]. This situation has been improved by realization of the value of detecting antibodies against cyclic citrullinated peptides and/or proteins (ACPA) in the identification of individuals likely to develop RA, even years before the manifestation of disease symptoms [6]. The presence of ACPA has been suggested to predict more severe disease [7-9]; however, not all RA patients are seropositive for ACPA, and in some patients diagnosis remains difficult, which can lead to delays in the initiation of the appropriate treatment. Several commercially available tests based on the detection of ACPA have been introduced, but they are problematic for diagnostic purposes, since they are not a prerequisite for the development of RA. Normally, to achieve a specificity of 98%, any test needs to have a sensitivity of <75%; hence their use can lead to a significant proportion of false-negative results [10].

Metabolic profiling (metabolomics) is an approach that could facilitate a more general and robust diagnosis and consequently an improved prognosis. Metabolic profiling has been used to identify biomarkers for several diseases [11]. The fundamental rationale in metabolomics is that perturbations in a biological system, for instance, those caused by a disease, will be detectable as changes in concentrations of certain metabolites. In some cases (for example, congenital metabolic diseases), it may be possible to identify a single, robust diagnostic metabolite, but in many cases the perturbations are more subtle, involving the activation of multiple enzymatic pathways. In such cases, it is unlikely that any single biomarker will be sufficiently specific for diagnostic purposes. However, by using multivariate statistics, it may be possible to describe patterns of biomarkers that are highly discriminatory for the perturbation and/or disease state [12,13]. An additional advantage of diagnosing patients using a metabolomic strategy is the possibility of revealing underlying biochemical phenomena associated with the disease, thus providing insights that help the development of a better understanding of the disease state.

The aim of this study was to assess the value of metabolic profiling in the diagnosis of patients with early RA by discriminating RA patients from healthy controls using multivariate analysis of the data for the measured metabolites. Patients with RA were also compared with psoriatic arthritis (PsoA) patients to evaluate the specificity of the metabolic changes in RA.

## Materials and methods

**Figure 1:**
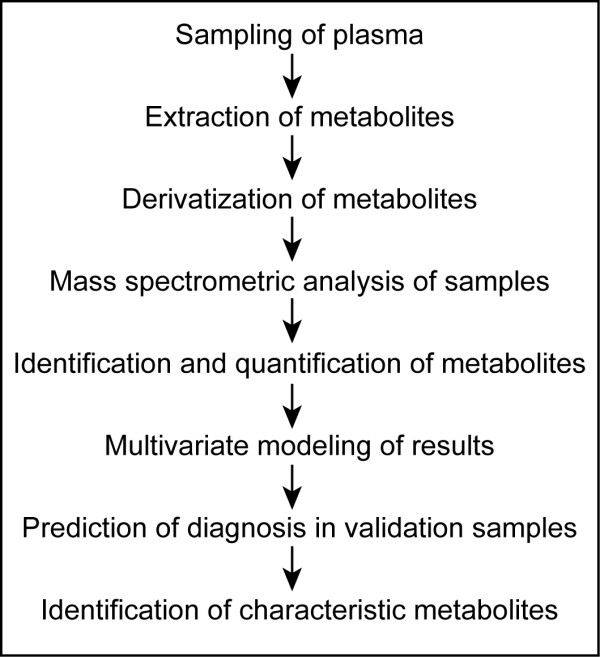
**Overview of the metabolic profiling study**. The figure lists the consecutive steps in this study that allowed the diagnosis of RA patients and the identification of discriminative metabolites. Details of analytical and data-processing procedures can be found in the supplementary information in Additional file 1.

An overview of the experimental setup in this study is presented in Figure 1. Details of the analytical and data-processing procedures are in the supplementary information in Additional file 1[14-20].

### Clinical sampling

#### Study 1

Blood samples were collected from 25 patients with RA according to the American College of Rheumatology (ACR) 1987 criteria [21] and from 20 patients fulfilling the Moll and Wright classification criteria [22], as well as the newly proposed CASPAR (Classification Criteria for Psoriatic Arthritis) classification criteria for established PsoA [23]. All patients were attending the Rheumatology Clinic at Umeå University Hospital, Umeå, Sweden. The corresponding serum samples were stored at -80°C until analysis. Of the RA patients, 17 (12 females and five males) had a disease duration of less than 1 year (mean, 6 months), with a mean age (± SD) of 51.1 (± 17.8) years (range, 18 to 76 years of age), and seven were ACPA-seropositive as analysed using the Diastat kit from Axis-Shield Diagnostics Limited (The Technology Park, Dundee, UK) for anti-cyclic citrullinated peptide 2 (anti-CCP2). The other eight RA patients (four females and four males) with established disease had a mean (± SD) disease duration of 18.8 (± 9.4) years and a mean age of 50.3 (± 17.6) years (range, 26 to 77 years of age), and all were ACPA-positive. Nine of the patients with early RA were treated with disease-modifying antirheumatic drugs (DMARDs) (five received methotrexate, three received sulfasalazine and one received hydroxychloroquine), as were two of the patients with established RA (one was receiving methotrexate together with a TNF inhibitor, and one was receiving methotrexate and hydroxychloroquine). Four patients in each of the early and established RA groups were prescribed corticosteroids (mean daily dose of 8.75 mg of prednisolone). The mean (± SD) Disease Activity Score 28 (DAS28) [24] for the patients was 4.06 (± 1.16) and ranged from 2.03 to 6.72.

All 20 patients (10 female and 10 male) with PsoA were seronegative for ACPA and rheumatoid factor (RF) and predominantly had peripheral arthritis. Three of the patients had mono- or oligoarthritis, and 17 patients had polyarthritis. Five of the patients also presented with axial disease. The mean (± SD) age of the PsoA patients was 48 (± 12) years (range, 31 to 68 years of age) with a mean (± SD) duration of 15 (± 12) years for joint disease and 20 (± 11) years for skin disease. Six of the patients were treated with DMARDs (three with methotrexate, two with cyclosporine and one with sulfasalazine), and five were prescribed nonsteroidal anti-inflammatory drugs.

Study 1 also included 10 healthy controls (eight female) recruited from the population register of the county of Västerbotten, Sweden, with a mean age of 55.2 years (range, 44 to 69 years).

Two different types of metabolite profiling were carried out, and full data were obtained for 21 of the RA patients, nine of the controls and 17 of the PsoA patients.

#### Validation study

The validation study was performed using separate RA and control cohorts collected at different global sites, thereby providing an assessment of the general applicability of the metabolite profiles. Four patients were attending the rheumatology department, Lund, Sweden, whilst 10 were from rheumatology departments in Russia (Institute of Rheumatology, City Clinical Hospital and Municipal Clinical Hospital, Moscow, and State Medical University, St. Petersburg).

All 14 RA patients (10 female and four male) fulfilled at least four criteria for RA as defined by the ACR [21], and the mean age of the group was 58.1 years (range, 45 to 76 years of age). Four patients were being treated with a combination of methotrexate and corticosteroids, three with methotrexate alone and two with corticosteroids. The mean (± SD) DAS28 [24] for this cohort of patients was 5.82 (± 0.74) and ranged between 4.50 and 7.00.

The 20 controls, all males, were healthy volunteers recruited through advertisement by the Karolinska University Hospital, Stockholm, Sweden. Their mean age was 26.0 years (range, 20 to 45 years of age).

Both Study 1 and the validation study were approved by the relevant regional ethics committees of the institutions involved, and all participants gave their written informed consent.

### Statistical analysis

#### Multivariate modeling

Chemometrics includes efficient and robust methods for the analysis of large multivariate data tables that produce interpretable and reliable prediction models. In biology, chemometric methodology has been largely overlooked in favour of traditional statistics. It has not been until recently that the overwhelming size and complexity of the 'omics' technologies have driven biology towards adopting chemometric analysis [13].

Orthogonal projections to latent structures-discriminant analysis (OPLS-DA) [18,20] is a multivariate classification technique used for finding patterns in large multivariate data sets that describe differences between the groups under study. The OPLS-DA model can map and separate all disease discriminating variation (predictive variation) from the variation that is uncorrelated to disease discrimination (orthogonal variation). Examples of orthogonal variation can be related to sex, age, sampling and experimental issues. OPLS-DA model interpretation is straightforward as the correlation (p(corr)) provides a direct measure of the discriminatory capability of each metabolite [25].

In this study, RA patients were compared with healthy controls and PsoA patients, and OPLS-DA models were used for (1) mapping the metabolic differences between the disease classes, (2) predicting disease in new individual samples, and (3) finding a specific metabolic pattern indicative of RA. Technical aspects of the OPLS algorithm (the basis for OPLS-DA) have been described fully by Trygg and Wold [18]. A technical description of OPLS-DA, together with application studies, has been provided by Bylesjö *et al. *[20].

Predicting the class of new individuals is a possible method for diagnosing RA. This may be achieved by fitting an OPLS-DA model to discriminate RA patients from controls. A binary response variable in which "1" indicated RA patients and "-1" indicated controls was created (see Keun *et al. *[26]). Each row represents a sample from one individual. Assignment of this binary variable has no influence on results, but for visual clarity it was decided to set the RA patients to positive values throughout the study.

The models were tested using a sevenfold cross-validation procedure in which one-seventh of all patients were omitted from the model fitting and subsequently predicted by the obtained model. This procedure is repeated seven times so that all patients are left out in turn from the modeling procedure [27]. Additional validation of the results was achieved by predicting the diagnosis of a group of patients in a separate study (the validation study) using the model fitted in study 1. Prediction of the excluded samples gives a numerical value that allows diagnosis. Separate models for healthy controls versus RA patients and PsoA patients versus RA patients were used.

All multivariate modeling was performed using Simca-P+ version 12 (Umetrics, Umeå, Sweden).

#### Univariate statistics

All statistical tests were two-sided Student's *t*-tests for samples with equal variance. Standard scores (*z*-scores) were calculated using the mean of the control group (healthy subjects or patients with PsoA) for comparisons between the groups. Only identified metabolites are presented in the plots and tables.

## Results

Combination of the data acquired using gas chromatography-mass spectrometry and liquid chromatography-mass spectrometry resulted in semiquantitative data being available for 267 (study 1) and 240 (validation study) putative metabolites. Eighty-three metabolites were assigned a positive identification in study 1 (see Additional file 1) and fifty-two were positively identified in both study 1 and the validation study.

The aim of this study was to evaluate the potential for diagnosing RA using metabolic profiling. For this purposed, OPLS-DA classification models were constructed for the discrimination between RA patients and healthy controls and between RA patients and PsoA patients, respectively. The results presented are predicted values based on multivariate models using all measured metabolites. The models are fitted using data from study 1, and the results are calculated using cross-validation (see Materials and methods) and data from the validation study.

**Figure 2:**
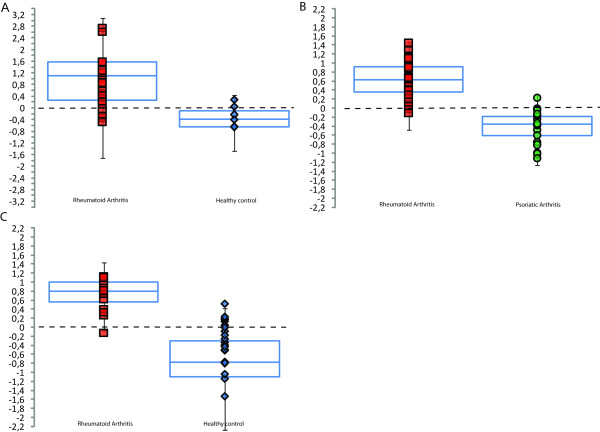
**Multivariate prediction of diagnosis of patients and healthy controls based on metabolite patterns**. **(A) **Box-and-whisker plot showing the prediction results from cross-validation in the study 1 rheumatoid arthritis (RA) versus control orthogonal projections to latent structures-discriminant analysis (OPLS-DA) model. Negative values correspond to controls and positive values indicate RA diagnoses (see Materials and methods). The blue outlined box denotes the median and 25% and 75% quartiles. The vertical black bars mark the median +1.5 times the interquartile range. **(B) **Prediction results for discrimination between RA and psoriatic arthritis patients using cross-validation. **(C) **Prediction results from the follow-up study. The model was built using study 1 data (52 metabolites and nine observations from each group).

Predictions based on classifying RA patients versus healthy controls in study 1 resulted in a sensitivity of 81% and a specificity of 67% (Figure 2A). RA patients and PsoA patients were classified with a sensitivity of 90% and a specificity of 94% (Figure 2B).

The most reliable method of validating the usefulness of metabolomic findings is to undertake a predictive approach in an entirely different patient cohort. Consequently, a validation study was carried out after evaluation of study 1 using a new group of patients. Fourteen patients with RA and 20 healthy controls had their blood metabolites profiled, and a diagnosis of RA was predicted using the model developed in study 1. The validation samples were obtained from different hospitals and analysed by different personnel, thereby giving a realistic indication of the diagnostic performance that may be expected in future applications. When comparing two different studies, it is necessary that the variables (metabolites) in each study be identical, thus the full metabolomic data set was not used in the predictions; instead, we used a subset of 52 metabolites that were identified and confirmed to be identical in the two studies. This validation resulted in a sensitivity of 93% and a specificity of 70% for RA (Figure 2C).

**Figure 3:**
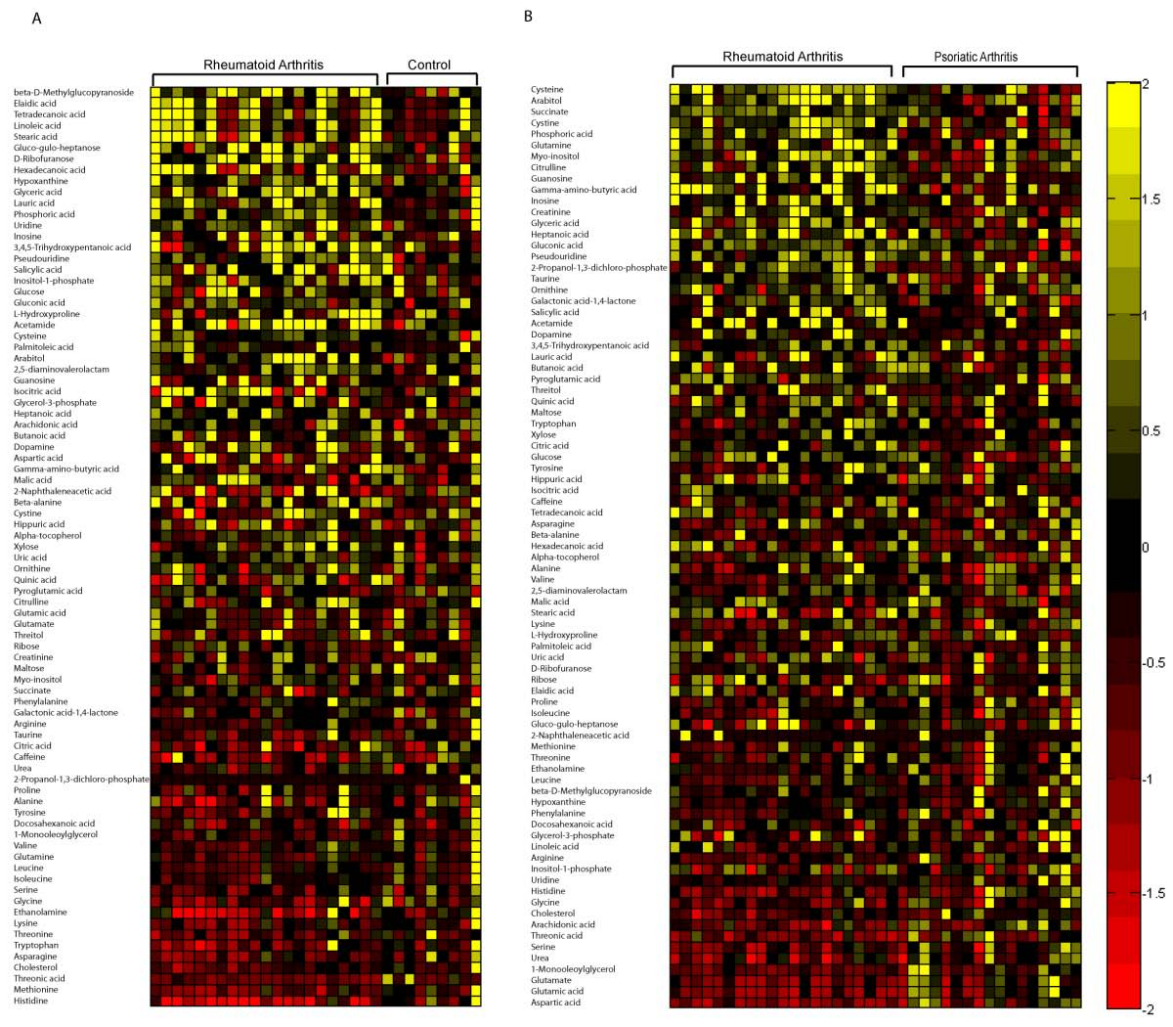
**Metabolic patterns seen in patients and healthy controls**. **(A) **Heat map showing the standard score for the each metabolite for each rheumatoid arthritis (RA) patient and each healthy control. The standard score shows how the concentration of each metabolite is related to the mean value of the control group for each individual. The standard score was calculated using the mean of the controls to attenuate the RA patients. Yellow color indicates that the metabolite is increased compared to the mean of the control group; red that it is decreased. Metabolites are sorted according to the correlation to RA in the RA versus control orthogonal projections to latent structures-discriminant analysis (OPLS-DA) model. The standard score is truncated to -2/2 for clarity. **(B) **Similar plot to Figure 3A, but showing RA and psoriatic arthritis (PsoA) patients. The standard score was calculated using the mean of the PsoA patients to attenuate the RA patients. Yellow color indicates that the metabolite is increased compared to the mean of the PsoA group; red that it is decreased. Metabolites are sorted according to the correlation to RA in the RA versus PsoA OPLS-DA model. The standard score is truncated to -2/2.

An additional aim of this study was the identification of metabolites that would be useful for discriminating between RA patients and controls. OPLS-DA models allow easy identification of those metabolites that are present in different concentrations in the groups being compared by generating correlation values for each metabolite. Plots showing the relative metabolite concentrations for each individual in the study are presented in Figures 3A and 3B. These plots are sorted according to the correlation of the metabolite to RA from the largest negative to the largest positive correlation. Metabolites found in statistically significantly different concentrations, according to Student's *t*-test, are listed in Table 1. A list of mean values and standard deviations for all identified metabolites is provided as supplementary information (Additional file 1). Prediction models built using only identified metabolites actually gave slightly better predictive results than the full model. In the case of study 1, the sensitivity was 86% and the specificity was 78%. This indicated that some of the unidentified metabolites were "experimental noise" and were not required for the diagnosis of RA.

**Table 1:** Metabolites found in different concentrations in RA patients and controls and in RA patients vs. PsoA patients^a^

RA relative to control	*P *value	RA relative to PsoA	*P *value
Increased		Increased	
	
Glyceric acid	0.029436	Glutamine	0.030049
D-ribofuranose	0.018080	Heptanoic acid	0.008232
Hypoxanthine	0.048579	Succinate	0.005375
		Pseudouridine	0.030814
		Inosine	0.032232
		Guanosine	0.023726
		Arabitol	0.004232
		Cystine	0.009069
		Cysteine	0.000118
		Phosphoric acid	0.010883
Decreased		Decreased	
	
Histidine	0.000109	Aspartic acid	0.000090
Threonic acid	0.000784	Glutamic acid	0.000248
Methionine	0.011428	Glutamate	0.001000
Cholesterol	0.023463	Histidine	0.007315
Asparagine	0.024166	Serine	0.008824
Threonine	0.046816	Arachidonic acid	0.038373
		Cholesterol	0.027836
		Threonic acid	0.002215
		1-Monooleoylglycerol	0.002369

^a^RA, rheumatoid arthritis; PsoA, psoriatic arthritis. *P *values are from the two-sided Student's *t*-test for samples with equal variance.

## Discussion

The aim of the present study was to assess the feasibility of employing metabolic profiling as a tool in the diagnosis of RA. The sensitivity of 93% is a good result compared with the tests currently available, which are based primarily on the detection of ACPA or RF. The specificity in the range of 70% to 80%, on the other hand, is not up to par with ACPA-based tests. Further appealing aspects of the metabolic profiling method were the capability of identifying individuals in both the early and advanced stages of RA disease and that this method was able to confirm the diagnosis of patients who were ACPA-negative and who would not have been diagnosed using the antibody tests. Metabolic profiling of biomarkers is therefore primarily a candidate method for closing some of the gaps in the diagnosis of RA that currently exist, especially with regard to ACPA-negative patients with early disease. Consequently, the prospects for patients without positive results in ACPA tests can be substantially improved.

It is important to note that the prediction of a diagnosis of RA was based on calculations using the full data set. The metabolites subsequently found to have the largest impact on the model are therefore the most important in reaching the diagnosis. Since the main result of the study is the diagnostic prediction, no correction for multiple statistical testing was made, but it was decided to report *P *values to illustrate the impact of each metabolite on the final result.

The cohorts in study 1 and in the validation study contained patients attending different clinics. It is encouraging that similar metabolic perturbations were detectable in RA patients from different geographical areas. This indicates that the metabolic perturbations seen are indeed related to RA disease processes, and further studies including larger patient and control cohorts are warranted to validate the biomarker profile for clinical use, to investigate its usefulness in prognosis and to investigate the various metabolic markers in relation to the disease process in RA.

A number of questions need further elucidation before clinical use of metabolic markers can become relevant. First, it is necessary to establish whether the metabolic profile is specific for RA or whether similar metabolic perturbations accompany other inflammatory diseases. To investigate this question, a cohort of patients with PsoA was included in this study. The results indicated larger differences between the RA patients and the PsoA patients than between the RA patients and healthy controls, as judged by between-group variability and the number of statistically significant metabolites differentiating the groups. This was somewhat surprising, considering the two inflammatory diseases. To arrive at a complete assessment of the usefulness of metabolic perturbations in the diagnosis of RA, any future study should include other groups of patients with a diagnosis other than RA, such as systemic lupus erythematosus, polymyalgia rheumatica and gout, as well as noninflammatory conditions, such as osteoarthritis.

It was perhaps surprising that better diagnostic results were achieved in the follow-up study than in study 1. A possible explanation is that these predictions were derived with fewer variables in the model. It is well known that variable selection can be a useful tool for optimising multivariate prediction models, since many of the detected variables (in this case, metabolites) carry no systematic information about the health status of the individual [28]; that is, they are the same in RA patients as in controls and only introduce noise in the model. As described above, better results were obtained in study 1 by decreasing the number of variables in the prediction model; however, the full data available are presented to give a more complete picture of the findings. Selection of the most discriminatory metabolites would almost certainly be required before using a metabolic marker profile in a clinical setting, since in these circumstances there would be a question of quantifying a limited number of metabolites for practical reasons. Similar diagnostic capability could be maintained with as few as seven metabolites (data not shown). The validation study was worse than study 1 in terms of matching the cases and controls, but the predictions were made on the basis of the results of study 1, indicating that the metabolites highlighted in study 1 were also relevant for distinguishing the individuals in the validation study.

Another question that needs clarification is how the metabolic profile of a patient is affected by disease activity and medical treatment. The RA patients classified as being healthy included the patient with the lowest DAS28 value (2.03), but the other two patients who were wrongfully classified as healthy had average DAS28 values (4.22 and 5.05). It is possible that fluctuations in disease activity might affect the metabolic profile of patients to some degree, but in this study high disease activity was not a prerequisite for diagnosis on the basis of metabolic profiling.

The present study was not designed to assess the relationship of metabolites with disease activity, but an obvious aim of any follow-up study would be to investigate whether and how DAS28 [24] is correlated to changes in the metabolite profile. This study did include patients on different DMARD regimens as well as non-DMARD-treated patients. Drug treatment did not introduce systematic problems in the metabolic profiling diagnosis; it is likely, however, that a number of metabolites are affected by treatment, and it would be interesting to map these effects further. It is even possible that the patient response to treatment could be correlated to changes in metabolic profile.

It is well known that factors such as sex, age, comorbidity, lifestyle and circadian rhythms [29-32] can affect the metabolic profile. This makes it important that the method be validated in larger studies accounting for these possible confounders before it is introduced clinically. Since it is difficult to correct for these factors, the main strategy would be to design studies that allow the assessment of potential conditions that could impair the diagnostic results of the method. In attempts to ensure that the profile found in this study was robust in terms of separating RA patients from controls, the validation study was performed using patients not attending the same hospital clinic. This gives some confidence that the metabolites identified are relevant to RA, but larger prospective studies will be the best way of identifying the potential role of metabolic profiling within the field of rheumatology.

One of the fields that has driven the development of metabolic profiling is systems biology, in which the aim is to gain an understanding of a disease by, for example, comparing perturbations in metabolites, proteins, gene transcripts and genes to those found in healthy individuals [12]. Since the present study is purely a study of metabolites, it is difficult to deduce the exact processes behind the observed perturbation between RA patients and controls. However, provided that these results can be reproduced, metabolic profiling may provide a starting point for mechanistic investigations into the pathogenesis of RA.

A decreased histidine level was one of the most specific metabolic markers for RA in this study. Previous findings have suggested that histidine catabolism is increased in patients with RA, although the reasons for this are unclear [33]. None of the direct products of histidine metabolism were detected in this study, but this should be the topic of a future study because of its apparent importance in RA and it might possibly lead to a better understanding of the pathophysiology of RA. PsoA patients also had higher histidine levels than RA patients, even though the difference between these groups was smaller than that between RA patients and controls.

Cholesterol is another compound with a well-established link to RA. The total cholesterol level in serum was measured in this study, therefore precluding the possibility of commenting on the specific composition of lipoprotein particles. Our results are consistent with an earlier report of lowered total cholesterol levels in RA patients with active inflammation [34]. Others have shown cholesterol levels in some groups of patients to be inversely correlated with disease activity and that the lipid composition in RA patients was generally unfavourable [35,36].

Threonic acid, a metabolite of ascorbic acid, was found to be significantly decreased. In the 1930s ascorbic acid was found to be reduced in the blood of RA patients, although there does not appear to be any clinical or pathological relevance to this observation [37].

Several amino acids were also slightly decreased in RA patients. Histidine, methionine, asparagine and threonine were found to be significantly decreased (*P *< 0.05), whilst several more showed a strong negative correlation to RA in the multivariate models. The compounds glyceric acid, D-ribofuranose and hypoxanthine were increased in RA patients. Taken together with positive correlations of uridine, pseudouridine and guanosine, this could indicate an increased rate of nucleotide synthesis, but the reasons for and relevance of this observation are hard to deduce. Furthermore, several free fatty acids showed a strong positive correlation to RA, even though this increase was not statistically significant.

An interesting topic for study in RA patients is the breakdown of arginine, particularly as both inducible nitric oxide synthases and arginase enzymes are known to exhibit increased activity in RA [38,39]. Only a moderate decrease of arginine and a slight increase of ornithine and citrulline were detected, but none of the effects were statistically significant. Although the exact correlation between most of the stronger metabolic markers for RA and the disease are unknown, it is proposed that metabolomics combined with other system biology techniques and appropriate mechanistic analysis can be a highly valuable tool for research into the processes underlying RA and other related diseases.

Both ACPA-positive and ACPA-negative patients participated in the study. This was mainly to ensure that the metabolic profiling method was applicable to both groups. No difference in the diagnostic properties of the method was detected between the two groups. When modeling ACPA-positive against ACPA-negative patients, the outcomes were weaker than those seen when modeling RA patients against controls. This indicates that though there are slight metabolic differences dependent on ACPA status, these are minor compared with the differences introduced by RA disease *per se*.

## Conclusions

This is the first time that the usefulness of metabolic profiling has been demonstrated in the diagnosis of RA. It appears that the method can be developed as a complementary diagnostic tool and is one that is especially applicable to patients who are seronegative for ACPA. Since the metabolic profile for individual patients is highly dynamic compared with, for example, genes and protein levels, it would be worth studying how these metabolites correlate with disease activity. It is possible that following the metabolite profile of individual patients could be a tool for predicting the disease course and thereby facilitate optimal treatment.

## Abbreviations

ACPA: antibodies against citrullinated peptides/proteins; DMARD: disease-modifying antirheumatic drug; OPLS-DA: orthogonal projection to latent structures-discriminant analysis; PCA: principal component analysis; PsoA: psoriatic arthritis; RA: rheumatoid arthritis; TNF-α: tumor necrosis factor-α.

## Competing interests

TL, TM and JT are shareholders of AcureOmics AB, where JG is both employed and a shareholder. CJS is employed by and a shareholder of Active Biotech Research AB. The authors declare no other competing interests. No financing has been received from these companies.

## Authors' contributions

RM participated in data acquisition and analysis, the conception and design of the study and wrote the manuscript. TL participated in the conception and design of the study. JG participated in the conception and design of the study. CJS participated in the acquisition of the data and revision of the manuscript. GMA participated in the conception and design of the study and acquisition of the data. SRD participated in the conception and design of the study, acquisition of the data and drafting of the manuscript. TM participated in the conception and design of the study and acquisition of the data. JT participated in the conception and design of the study, data analysis and revision of the manuscript.

## Supplementary Material

Additional file 1 **Supplementary Information**. This document contains supplementary information on the technical aspects of the metabolic profiling analysis. The document also contains a list of mean values and standard deviation for identified metabolites in study 1. Click here for file
