# Supplementary material for: Diagnostic properties of metabolic perturbations in rheumatoid arthritis
Source: Arthritis Res Ther. 2011 Feb 8;13(1):R19. doi: 10.1186/ar3243 (PMC3241363; doi:10.1186/ar3243)
Supplement: Additional file 1 — Supplementary Information. This document contains supplementary information on the technical aspects of the metabolic profiling analysis. The document also contains a list of mean values and standard deviation for identified metabolites in study 1. [file ar3243-S1.DOC]

**Supplementary Information**

# Metabolic profiling

# *Metabolite extraction*

Extraction of metabolites from plasma was undertaken following the protocol of A *et al*., using an extraction mixture of methanol : water (9:1), containing 11 internal standard compounds. The stable isotope reference compounds, [^2^H_4_]-succinic acid, [^13^C_5_,^15^N]-glutamic acid, [^2^H_7_]-cholesterol, [1,2,3-^13^C_3_]-myristic acid, [^13^C_5_]-proline, and [^13^C_4_]-disodium 2-oxoglutarate were purchased from Cambridge Isotope Laboratories (Andover, MA), [^13^C_6_]- glucose, [^13^C_12_]-sucrose,[^13^C_4_]-hexadecanoic acid, and [^2^H_4_]-1,4-butane-diamine-2HCl, were from Campro (Veenendaal,The Netherlands), and 2-hydroxy-[^2^H_6_]-benzoic acid was from Icon (Summit, NJ).

### *GC-MS analysis*

An aliquot, 100 µL, of thawed plasma was added to Sarstedt safety cap tubes and 900 µL of the extraction mixture consisting of methanol:water (9:1) containing 11 isotopically labeled internal standards were added. The mixture was shaken for 2 min at 30 Hz and stored in an ice-bath for 2 hours before centrifugation in an Eppendorf centrifuge (Model 5417C) for 10 min at 4ºC and14000 rpm. 200 µL of the supernatant were transferred to GC-MS-vials and evaporated to dryness, using a Speedvac. The samples were then derivatized by shaking them for 10 min with 30 µL pyridine containing methoxyamine, 15 µg/µL, followed by incubation at 70ºC for 60 min. Following incubation at RT for 16 hours N-Methyl-N-trifluoroacetamide (MSTFA), 30 µL, containing 1 % Trimethylchlorosilane (TMCS) was added. The mixture was vortex-mixed and allowed to react for 1 hour before addition of 30 µL of heptane containing 15 ng/µL methyl stearate. The extracted and derivatized samples were placed in a Agilent 7683 Series auto sampler (Agilant, Atlanta, GA) and 1 µL was injected splitless into a Agilent 6980 GC equipped with a 10 m x 0.18 mm i.d. fused-silica capillary column chemically bonded with 0.18 Tm DB5-MS stationary phase (J&W Scientific, Folsom, CA) coupled to a Pegasus III TOFMS (Leco Corp., St Joseph, MI) mass spectrometer. The injector temperature was 270ºC, the septum purge flow rate was 20 ml min^-1^ and the purge was turned on after 60 s. The gas flow rate through the column was 1 ml min^-1^, the column temperature was held at 70ºC for 2 minutes, then increased by 40ºC min^-1^ to 320ºC, and held there for 2 min. Ions were generated by a 70 eV electron beam at an ionization current of 2.0 mA, and 30 mass spectra s^-1^ were recorded in the mass range from m/z 50 to 800, after a solvent delay of 170 s. The ion source was maintained at 200ºC.

### *UPLC-MS amino acid analysis*

The extraction procedure for amino acid analysis was done analogous to that for GC-MS analysis. Following incubation with the extraction mixture and centrifugation an aliquot of the supernatant, 150 µL, was evaporated to dryness using a Speedvac. Derivatisation of amino acids was achieved using the AccQ-Tag kit obtained from Waters (Millford, MA) as specified in the manufacturer’s instructions. Analysis was on a Waters Acquity UPLC system coupled to a Micromass LCT Premier mass spectrometer (Waters, Millford, MA) operated in W-mode. Amino acids were quantified using peak area.

## Data Processing GC-MS

All non-processed MS-files from the metabolic analysis were exported from the ChromaTOF software in NetCDF format to MATLAB™ software 2006b (Mathworks, Natick, MA, USA), in which all data pre-treatment procedures, such as base-line correction chromatogram alignment, data compression and Hierarchical Multivariate Curve Resolution (H-MCR) were performed using custom scripts as described by Jonsson *et al.* . All manual integrations were performed using ChromaTOF 2.12 software (Leco Corp., St Joseph, MI, USA) or in-house MATLAB scripts. The data processing protocols resulted in peak areas for the derivatized metabolites and corresponding mass spectra.

*Metabolite libraries and metabolite identification*

The metabolites were identified by comparison of retention indices and mass spectra with data in commercial, as well as in-house, retention indexes and mass spectra libraries using NIST MS Search 2.0 (National Institute of Standards and Technology, 2001).

*Quantification of identified metabolites*

The data processing of the GC-MS data using the H-MCR script resulted in initial datasets. All variables were checked manually and variables originating from internal standards and processing artifacts excluded. Additionally, chromatographic peaks originating from one compound but split during the data processing were re-processed using the H-MCR program or by manual integration. For study 1 GC-MS and UPLC-MS datasets were combined before modeling and doublet metabolites were removed. In all, semi quantitative data was achieved for 267 (study 1) / 240 (Validation study) putative metabolites.

## *Sample normalization*

### GC-TOF-MS

The dataset was normalized with the aid of the 11 added internal standards; a non-centered principal component analysis (PCA) model was built on the basis of the intensity of selected ions originating from the internal standard compounds. The magnitude of the PCA model t1-score of a given sample was taken as a general measure of the intensity. Differences in intensity between samples are primarily expected to originate from differences in the system inlet efficiency. These effects are removed by dividing all measured metabolite intensities with the PCA model score t1-value for the corresponding sample.

### UPLC-MS

Normalization was done by dividing all integrated areas with the value for the internal reference compound glutamic acid.

In order to assign samples from study 1 and the follow-up study comparable variance values these were normalized using quantile normalization before building the RA vs. healthy control model.

## *Orthogonal Projections to Latent Structures (OPLS) and classification (OPLS-DA)*

OPLS is an extension to the supervised PLS regression method. In cases where the study object is described by qualitative traits, such as a disease diagnosis, OPLS can, analogously to PLS-DA, be used for discrimination (OPLS-DA) . In OPLS-DA, all observations predicted by the model are assigned a class-specific numerical value, ŷ, where a zero threshold was employed to determine the appropriate class. However, if the classes are heterogeneous, or differ greatly in size, this may not be an optimal method of assigning class membership, and an alternative approach is provided by Bylesjö *et al.* Further details of the OPLS algorithm are described.

In OPLS two class data is modeled by the model:


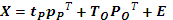
 (2a)


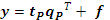
 (2b)

In OPLS-DA, the predictive component loading (
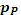
) provides a direct measure of the influence of each variable. In metabolomics studies, the correlation scaled loading values reveal the most discriminatory variables. Correlation scaled p(corr) loading values are calculated as:

,𝑝-_𝑖_.,𝑐𝑜𝑟𝑟.= ,,**𝒕**-𝑇.,**𝑿**-𝑖.-(**,𝒕.,,𝑿-**𝑖**..**). (3)

X_i_ represents the i^th^ variable in the X matrix (quantified metabolites).

The amount of explained variance in a model was calculated using the formula:


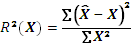
 (4)


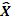
 being the values of **X** predicted by the model. Analogue calculations can be made for the **Y** matrix. Model complexity was determined using cross-validation.

*Classification*

The diagnostic capability of the metabolites detected was therefore tested using the OPLS-DA model, a pattern recognition technique used for finding subtle class differences in multivariate datasets. In study 1 classification was done using cross-validation (see methods) and the results shown are the results from the cross-validation round where the samples were not included in model building. In all cases binary a dummy matrix, in which the value 1 was assigned to RA patients and -1 to controls, was used for building the OPLS-DA classification model.

**List of Mean-values and standard deviation for identified metabolites – Study 1**

Since the data is recorded using two different analytical platforms and is semi-quantitative it is not informative to do direct comparisons between individual metabolites in this list.

|  | **Mean Global** | **Standard deviation - Global** | **Mean control** | **Standard deviation - Control** | **Mean RA** | **Standard deviation - RA** | **Mean PsoA** | **Standard deviation - PsoA** |
| --- | --- | --- | --- | --- | --- | --- | --- | --- |
| **1-Monooleoylglycerol** | 1,292E-01 | 4,897E-02 | 1,659E-01 | 6,655E-02 | 1,203E-01 | 3,810E-02 | 1,206E-01 | 4,387E-02 |
| **2,5-diaminovalerolactam** | 1,174E+00 | 3,124E-01 | 1,269E+00 | 2,689E-01 | 1,149E+00 | 3,206E-01 | 1,155E+00 | 3,312E-01 |
| **2-Naphthaleneacetic acid** | 1,988E+03 | 1,176E+03 | 4,921E+05 | 1,554E+06 | 9,329E+04 | 2,792E+05 | 2,540E+05 | 1,061E+06 |
| **2-Propanol, 1,3-dichloro-phosphate** | 5,587E-02 | 2,108E-02 | 5,543E-02 | 2,480E-02 | 5,061E-02 | 1,259E-02 | 6,259E-02 | 2,622E-02 |
| **3,4,5-Trihydroxypentanoic acid** | 3,620E-02 | 2,587E-02 | 2,269E-02 | 6,190E-03 | 2,482E-02 | 1,044E-02 | 5,741E-02 | 3,191E-02 |
| **Acetamide** | 8,428E+03 | 3,813E+03 | 1,394E+05 | 2,578E+05 | 1,282E+04 | 1,725E+04 | 6,852E+04 | 1,821E+05 |
| **Alanine** | 1,588E-02 | 1,323E-02 | 1,211E-02 | 2,550E-03 | 1,720E-02 | 1,819E-02 | 1,623E-02 | 8,732E-03 |
| **Alpha-tocopherol** | 7,651E-02 | 3,591E-02 | 7,588E-02 | 3,260E-02 | 8,701E-02 | 3,983E-02 | 6,389E-02 | 2,964E-02 |
| **Arabitol** | 2,151E+05 | 5,330E+04 | 2,736E+05 | 1,001E+05 | 1,939E+05 | 4,312E+04 | 2,336E+05 | 8,293E+04 |
| **Arachidonic acid** | 1,619E-01 | 1,283E-01 | 1,090E-01 | 7,377E-02 | 1,068E-01 | 6,609E-02 | 2,580E-01 | 1,541E-01 |
| **Arginine** | 1,601E+00 | 3,627E-01 | 1,834E+00 | 5,044E-01 | 1,641E+00 | 2,967E-01 | 1,429E+00 | 2,770E-01 |
| **Asparagine** | 5,663E-01 | 2,128E-01 | 6,195E-01 | 2,084E-01 | 4,961E-01 | 1,715E-01 | 6,250E-01 | 2,448E-01 |
| **Aspartic acid** | 4,055E-02 | 1,407E-02 | 5,095E-02 | 1,209E-02 | 3,296E-02 | 9,083E-03 | 4,442E-02 | 1,550E-02 |
| **Beta-alanine** | 5,416E-01 | 1,742E-01 | 5,975E-01 | 2,869E-01 | 5,099E-01 | 1,163E-01 | 5,512E-01 | 1,611E-01 |
| **Beta-D-Methylglucopyranoside** | 1,030E+00 | 3,159E-01 | 1,124E+00 | 4,634E-01 | 9,698E-01 | 2,161E-01 | 1,056E+00 | 3,327E-01 |
| **Butanoic acid** | 2,746E-02 | 1,393E-02 | 2,215E-02 | 7,821E-03 | 2,852E-02 | 1,274E-02 | 2,895E-02 | 1,746E-02 |
| **Caffeine** | 1,736E-01 | 3,810E-02 | 1,926E-01 | 5,010E-02 | 1,659E-01 | 2,966E-02 | 1,730E-01 | 3,923E-02 |
| **Cholesterol** | 1,296E-01 | 5,887E-02 | 1,742E-01 | 7,818E-02 | 1,146E-01 | 4,275E-02 | 1,244E-01 | 5,640E-02 |
| **Citric acid** | 9,370E-02 | 2,997E-02 | 9,027E-02 | 2,969E-02 | 9,982E-02 | 3,050E-02 | 8,794E-02 | 2,978E-02 |
| **Citrulline** | 6,608E-01 | 2,767E-01 | 6,134E-01 | 2,945E-01 | 6,330E-01 | 2,147E-01 | 7,202E-01 | 3,363E-01 |
| **Creatinine** | 3,178E-01 | 1,043E-01 | 3,276E-01 | 1,194E-01 | 2,776E-01 | 6,575E-02 | 3,624E-01 | 1,202E-01 |
| **Cysteine** | 4,390E-01 | 1,634E-01 | 5,463E-01 | 2,208E-01 | 4,096E-01 | 1,365E-01 | 4,185E-01 | 1,449E-01 |
| **Cystine** | 3,396E-01 | 1,358E-01 | 4,369E-01 | 1,568E-01 | 3,239E-01 | 1,322E-01 | 3,074E-01 | 1,101E-01 |
| **D-Ribofuranose** | 2,588E+05 | 7,532E+04 | 3,614E+05 | 1,116E+05 | 3,562E+05 | 1,834E+05 | 3,399E+05 | 1,403E+05 |
| **Docosahexanoic acid** | 3,611E-01 | 1,398E-01 | 3,908E-01 | 1,528E-01 | 3,586E-01 | 1,493E-01 | 3,484E-01 | 1,263E-01 |
| **Dopamine** | 1,493E+00 | 3,496E-01 | 1,571E+00 | 4,924E-01 | 1,450E+00 | 2,784E-01 | 1,505E+00 | 3,568E-01 |
| **Elaidic acid** | 1,701E-01 | 8,575E-02 | 2,105E-01 | 8,209E-02 | 1,801E-01 | 7,354E-02 | 1,365E-01 | 9,361E-02 |
| **Ethanolamine** | 6,817E-01 | 2,611E-01 | 6,908E-01 | 2,397E-01 | 6,486E-01 | 2,177E-01 | 7,177E-01 | 3,243E-01 |
| **GABA** | 1,064E+00 | 9,391E-02 | 1,042E+00 | 3,540E-02 | 1,086E+00 | 1,223E-01 | 1,050E+00 | 7,013E-02 |
| **Galactonic acid-1,4-lactone** | 1,517E+06 | 2,132E+06 | 1,095E+06 | 9,754E+05 | 2,045E+06 | 2,989E+06 | 1,089E+06 | 8,720E+05 |
| **Gluco-gulo-heptanose** | 4,463E+04 | 1,507E+04 | 6,218E+04 | 2,298E+04 | 6,012E+04 | 1,656E+04 | 5,807E+04 | 2,023E+04 |
| **Gluconic acid** | 1,483E+05 | 1,133E+05 | 1,358E+05 | 1,537E+05 | 1,903E+05 | 1,167E+05 | 1,031E+05 | 5,917E+04 |
| **Glucose** | 5,533E+05 | 2,911E+04 | 5,659E+05 | 1,778E+04 | 5,624E+05 | 1,709E+04 | 5,355E+05 | 3,713E+04 |
| **Glutamate** | 2,935E+06 | 1,204E+06 | 2,893E+06 | 1,028E+06 | 2,911E+06 | 1,005E+06 | 2,987E+06 | 1,540E+06 |
| **Glutamic acid** | 3,882E+05 | 9,938E+04 | 3,876E+05 | 1,260E+05 | 3,554E+05 | 8,060E+04 | 4,291E+05 | 9,565E+04 |
| **Glutamine** | 2,366E+06 | 2,726E+05 | 2,319E+06 | 3,033E+05 | 2,474E+06 | 2,297E+05 | 2,257E+06 | 2,690E+05 |
| **Glyceric acid** | 6,691E+06 | 2,086E+06 | 5,607E+06 | 1,813E+06 | 7,433E+06 | 2,066E+06 | 6,347E+06 | 2,005E+06 |
| **Glycerol-3-phosphate** | 7,021E+04 | 2,099E+04 | 7,140E+04 | 2,514E+04 | 8,044E+04 | 1,484E+04 | 5,695E+04 | 1,871E+04 |
| **Glycine** | 7,795E+05 | 2,487E+05 | 6,796E+05 | 2,784E+05 | 7,992E+05 | 1,776E+05 | 8,082E+05 | 3,044E+05 |
| **Guanosine** | 2,245E+05 | 5,130E+04 | 2,192E+05 | 4,060E+04 | 2,264E+05 | 4,851E+04 | 2,251E+05 | 6,148E+04 |
| **Heptanoic acid** | 1,677E+05 | 3,894E+05 | 4,408E+04 | 1,632E+04 | 3,146E+05 | 5,539E+05 | 5,166E+04 | 3,269E+04 |
| **Hexadecanoic acid** | 1,628E+06 | 7,899E+04 | 1,651E+06 | 9,842E+04 | 1,637E+06 | 6,628E+04 | 1,604E+06 | 8,124E+04 |
| **Hippuric acid** | 1,013E+03 | 8,945E+02 | 1,877E+03 | 1,527E+03 | 5,875E+02 | 3,178E+02 | 1,080E+03 | 5,884E+02 |
| **Histidine** | 9,520E+05 | 5,064E+05 | 1,082E+06 | 5,132E+05 | 1,044E+06 | 4,571E+05 | 7,694E+05 | 5,370E+05 |
| **Hypoxanthine** | 5,135E+04 | 3,291E+04 | 3,864E+04 | 1,882E+04 | 3,845E+04 | 1,784E+04 | 7,401E+04 | 4,104E+04 |
| **Inosine** | 1,183E+05 | 8,341E+04 | 1,860E+05 | 1,683E+05 | 7,836E+04 | 1,180E+05 | 1,341E+05 | 1,442E+05 |
| **Inositol-1-phosphate, myo-** | 1,978E+05 | 1,702E+05 | 1,968E+05 | 1,218E+05 | 2,317E+05 | 2,125E+05 | 1,566E+05 | 1,270E+05 |
| **Isocitric acid** | 1,781E+05 | 1,216E+05 | 1,255E+05 | 1,000E+05 | 2,166E+05 | 1,395E+05 | 1,584E+05 | 9,613E+04 |
| **Isoleucine** | 1,189E+05 | 5,032E+04 | 1,050E+05 | 1,730E+04 | 1,301E+05 | 4,271E+04 | 1,124E+05 | 6,733E+04 |
| **Lauric acid** | 7,116E+04 | 2,219E+04 | 7,840E+04 | 2,936E+04 | 7,354E+04 | 1,638E+04 | 6,439E+04 | 2,375E+04 |
| **Leucine** | 6,571E+03 | 2,031E+03 | 6,068E+03 | 1,602E+03 | 6,623E+03 | 2,218E+03 | 6,774E+03 | 2,057E+03 |
| **L-Hydroxyproline** | 5,661E+03 | 2,318E+03 | 5,590E+03 | 1,801E+03 | 6,028E+03 | 2,557E+03 | 5,245E+03 | 2,298E+03 |
| **Linoleic acid** | 1,503E+05 | 6,114E+04 | 1,441E+05 | 4,185E+04 | 1,579E+05 | 6,852E+04 | 1,443E+05 | 6,218E+04 |
| **Lysine** | 2,121E+06 | 6,209E+05 | 2,332E+06 | 4,847E+05 | 2,163E+06 | 5,905E+05 | 1,959E+06 | 7,068E+05 |
| **Malic acid** | 2,129E+05 | 9,371E+04 | 1,934E+05 | 2,516E+04 | 2,213E+05 | 7,232E+04 | 2,128E+05 | 1,345E+05 |
| **Maltose** | 8,216E+04 | 4,556E+04 | 8,280E+04 | 3,523E+04 | 7,178E+04 | 5,189E+04 | 7,869E+04 | 4,319E+04 |
| **Methionine** | 1,684E+05 | 1,154E+05 | 2,266E+05 | 1,088E+05 | 1,665E+05 | 1,219E+05 | 1,399E+05 | 1,049E+05 |
| **Myo-inositol** | 1,450E+05 | 1,185E+05 | 8,423E+04 | 6,612E+04 | 1,710E+05 | 1,266E+05 | 1,449E+05 | 1,231E+05 |
| **Ornithine** | 7,657E+04 | 5,161E+04 | 8,044E+04 | 4,026E+04 | 8,327E+04 | 5,745E+04 | 6,624E+04 | 5,040E+04 |
| **Palmitoleic acid** | 3,814E+05 | 1,154E+05 | 3,801E+05 | 1,086E+05 | 4,137E+05 | 8,897E+04 | 3,423E+05 | 1,397E+05 |
| **Phenylalanine** | 9,108E+03 | 2,485E+03 | 9,769E+03 | 2,908E+03 | 9,742E+03 | 2,238E+03 | 7,974E+03 | 2,266E+03 |
| **Phosphoric acid** | 1,132E+06 | 5,995E+05 | 8,019E+05 | 2,551E+05 | 1,188E+06 | 4,201E+05 | 1,237E+06 | 8,384E+05 |
| **Proline** | 2,858E+05 | 3,472E+05 | 2,134E+05 | 3,599E+05 | 3,251E+05 | 3,506E+05 | 2,756E+05 | 3,507E+05 |
| **Pseudouridine** | 7,169E+04 | 3,273E+04 | 5,634E+04 | 1,622E+04 | 7,844E+04 | 3,703E+04 | 7,149E+04 | 3,229E+04 |
| **Pyroglutamic acid** | 1,163E+04 | 1,692E+03 | 1,114E+04 | 2,615E+03 | 1,177E+04 | 1,403E+03 | 1,171E+04 | 1,479E+03 |
| **Quinic acid** | 9,652E+03 | 5,371E+03 | 6,684E+03 | 2,803E+03 | 9,816E+03 | 4,942E+03 | 1,102E+04 | 6,443E+03 |
| **Ribose** | 2,759E+05 | 1,430E+05 | 2,635E+05 | 9,785E+04 | 2,591E+05 | 8,514E+04 | 2,643E+05 | 1,014E+05 |
| **Salicylic acid** | 5,248E+04 | 3,758E+04 | 3,321E+04 | 2,482E+04 | 5,851E+04 | 3,854E+04 | 5,524E+04 | 4,040E+04 |
| **Serine** | 1,375E+06 | 5,513E+05 | 1,035E+06 | 2,671E+05 | 1,484E+06 | 6,616E+05 | 1,421E+06 | 4,563E+05 |
| **Stearic acid** | 2,341E+04 | 1,436E+05 | 1,110E+05 | 3,285E+05 | 3,227E+03 | 2,723E+03 | 1,991E+03 | 2,356E+03 |
| **Succinate** | 5,687E+04 | 1,676E+04 | 5,168E+04 | 1,938E+04 | 6,312E+04 | 1,520E+04 | 5,189E+04 | 1,544E+04 |
| **Taurine** | 1,764E+05 | 5,964E+04 | 1,498E+05 | 6,088E+04 | 1,652E+05 | 4,635E+04 | 2,043E+05 | 6,557E+04 |
| **Tetradecanoic acid** | 3,393E+04 | 3,810E+04 | 4,440E+04 | 7,677E+04 | 2,338E+04 | 1,785E+04 | 4,141E+04 | 2,369E+04 |
| **Threitol** | 2,382E+05 | 5,180E+04 | 2,107E+05 | 5,159E+04 | 2,411E+05 | 5,232E+04 | 2,492E+05 | 4,894E+04 |
| **Threonic acid** | 8,356E+04 | 2,979E+04 | 9,414E+04 | 3,449E+04 | 7,858E+04 | 2,389E+04 | 8,410E+04 | 3,387E+04 |
| **Threonine** | 4,268E+04 | 4,169E+04 | 3,839E+04 | 3,333E+04 | 2,511E+04 | 1,519E+04 | 6,667E+04 | 5,590E+04 |
| **Tryptophan** | 9,006E+03 | 1,170E+04 | 8,604E+03 | 8,179E+03 | 1,316E+04 | 1,417E+04 | 4,089E+03 | 7,802E+03 |
| **Tyrosine** | 1,482E+04 | 2,886E+03 | 1,438E+04 | 3,159E+03 | 1,496E+04 | 2,508E+03 | 1,487E+04 | 3,309E+03 |
| **Urea** | 3,692E+04 | 7,294E+03 | 4,031E+04 | 1,037E+04 | 3,412E+04 | 3,969E+03 | 3,857E+04 | 7,736E+03 |
| **Uric acid** | 1,814E+04 | 1,111E+03 | 1,806E+04 | 7,429E+02 | 1,845E+04 | 9,759E+02 | 1,780E+04 | 1,351E+03 |
| **Uridine** | 5,113E+04 | 1,539E+04 | 6,216E+04 | 1,292E+04 | 7,236E+04 | 3,945E+04 | 6,374E+04 | 2,674E+04 |
| **Valine** | 1,951E+02 | 8,923E+01 | 2,139E+02 | 6,026E+01 | 2,258E+02 | 8,928E+01 | 1,471E+02 | 8,504E+01 |
| **Xylose** | 6,573E+04 | 3,055E+04 | 9,259E+04 | 9,059E+04 | 7,563E+04 | 6,116E+04 | 8,131E+04 | 7,176E+04 |
